# Genetic diversity and verification of plant material compliance of Cocoa (***Theobroma cacao L***.) in the Barombi-Kang Regional variety trial

**DOI:** 10.1371/journal.pone.0322169

**Published:** 2025-04-24

**Authors:** Nto Marie Claire Eyango, Olivier Sounigo, Olivier Fouet, Honoré Tekeu, François Pierre Djocgoué, Mousseni Ives Bruno Efombagn, Claire Lanaud

**Affiliations:** 1 Department of Plant Biology, Faculty of Science, University of Yaoundé I, Yaoundé, Cameroon; 2 Plant Pathology Laboratory, Institute of Agricultural Research for Development, Yaoundé, Cameroon; 3 Genetic Improvement and Adaptation of Mediterranean and tropical plants, Center for International Cooperation in Agricultural Research for Development Palmira, Valle Del Cauca, Colombia; 4 Biological Systems Department, Genetic Improvement and Adaptation of Mediterranean and tropical plants Institute, Center for International Cooperation in Agricultural Research for Development, Montpellier, France; Institute of Mediterranean Forest Ecosystems of Athens, GREECE

## Abstract

Cocoa (*Theobroma cacao L.)* is a pivotal agricultural commodity in Cameroon, which ranks as one of the top five global cocoa producers. This study focused on evaluating the genetic diversity and verifying the plant material compliance of cocoa genotypes in the Barombi-Kang Regional variety trial, employing 12 highly polymorphic SSR markers. A comprehensive analysis of 318 hybrid families and 15 parental genotypes was conducted, which revealed extensive genetic variability. The study found an average polymorphic information content (PIC) of 0.72 for hybrids and 0.68 for parents, alongside observed heterozygosity rates of 0.54 and 0.42, respectively, indicating a rich genetic reservoir. Significantly, an 18.55% labeling error rate was identified, underscoring prevalent issues in germplasm management that could impact the efficacy of breeding programs. These errors highlight the critical need for enhanced genetic verification protocols to ensure the accuracy and reliability of plant materials used in breeding. The genetic analysis also demonstrated substantial allelic richness with the hybrids showing an average of 72 alleles per locus, suggesting a high capacity for selection within the breeding pool. The data from this study not only reinforce the potential for genetic improvement of cocoa in Cameroon but also provide crucial insights into the genetic structure and population dynamics within the trial. Addressing the genetic and management challenges identified could lead to the development of superior cocoa varieties, enhancing yield, disease resistance, and environmental stress tolerance, thereby contributing to the sustainable advancement of the cocoa industry in Cameroon and beyond.

## Introduction

The cocoa tree (*Theobroma cacao L.*) is a perennial diploid plant (2n = 20) belonging to the Malvaceae family [1] It is predominantly an outcrossing species with a relatively small genome (430Mb) and thrives between 15° South and 20° North latitudes. Widely cultivated by small-scale farmers in the humid tropics as a cash crop [2–4], cocoa significantly contributes to the economies of many countries globally. Its beans are the primary raw materials for various products in the food, pharmaceutical, and cosmetics industries. Notably, cocoa has been recognized for its antioxidant properties, cardiovascular health benefits, and anti-tumoral effects [5]. It is believed to have originated from the Amazon forest, where it exhibits considerable genetic biodiversity [6–8].

Accounting for 6% of global production [9], Cameroon produces 262,112 tonnes of cocoa beans annually, making it the fifth-largest producer worldwide. Cocoa exports represent 58.7% of agricultural revenue and are the second-largest source of foreign exchange in the country, accounting for 12.5% of exports. The cocoa sector supports roughly 3 million people in Cameroon, either directly or indirectly.

Cocoa was introduced to Cameroon between 1876 and the first breeding program was implemented in the 1950s, focusing on clonal selection. A new program began in the early 1960s aimed at selecting high-yield varieties, and offspring from various crosses were planted and evaluated in the field at research stations in Nkoemvone, Southern Cameroon, and Barombi-Kang, Southwest Cameroon. Understanding the genetic diversity and population structure of cocoa collections is crucial for crop improvement. Historically, breeding efforts in Cameroon have focused on local cocoa material collected from farmers’ fields, including SNK accessions selected for their high yield potential The next phase involved the creation of seed fields by crossing local genotypes with introduced foreign ones, focusing mainly on those with potential for high yields. The breeding methods adopted over the past two decades have included a participatory approach, involving farmers in identifying high-yielding trees in their plantations and selecting new varieties during nursery trials. The current breeding program aims to select new hybrid and clonal varieties resistant to diseases and pests (mainly Phythophthora megakarya and mirids) and to begin searching for higher-quality varieties to add value to the product. Furthermore, Cameroon intends to introduce more genetic material to expand the useful genetic diversity in the gene bank, which is currently very limited. These trials exhibit broad genetic variability and distinct geographic origins chosen for their disease resistance and productivity.

However, as with many clonal propagation trials, associated errors tend to accumulate over time [10]. Historically, the characterization of genetic material from collections was limited to morphological and agronomic traits [11,12]. Studies have emphasized the importance of removing duplicates during the characterization of genetic material for breeding activities. Moreover, SSR markers are preferred for analyzing genetic diversity due to their abundance in the genome, high polymorphism, and codominant nature, making them suitable for characterizing heterozygous plants like the cocoa tree. The collections were assessed, described and characterized using molecular markers based on population structure and genetic diversity [8,13–15]. This study aims not only to evaluate labeling errors within the population but also to explore the level of genetic diversity of cocoa accessions cultivated in Cameroon.

## Materials and methods

### Plant material

The study included 360 cocoa genotypes encompassing the three main genetic groups of the species: Trinitario (Tr), Upper Amazonian Forastero (UAF), and Lower Amazonian Forastero (LAF). These consisted of 318 UAF ×Tr hybrid types from the Barombi-Kang regional variety trial belonging to eight full-sibling families, 15 parent genotypes from the clonal collection of the Agricultural Research Institute for Development (IRAD); of these parents, eight are used as male parents, eight as female parents. However, one is used as both male and female parents in different crosses, and 27 reference genotypes that are not present in Cameroon (Table 1).

**Table 1:** Hybrid Families; Parents and Reference genotypes used in the study.

Progeny Id	Geographic origin	Morpho-geographic group
UPA 134x SNK 64 (F2)	Cameroon	UAF x Tr
SNK 614 x SCA 24 (F6)	Cameroon	UAF x Tr x UAF
SNK 625 x NA 33 (F11)	Cameroon	UAF x UAF
T 60/887x ICS 89 (F14)	Côte d’Ivoire	UAF x Tr
SNK 12 x PA 150 (F15)	Côte d’Ivoire	Tr x UAF
T 60/78x T 85/87 (F17)	Ghana	UAF x UAF
MAN 15/2x T 85/799 (F19)	Ghana	LAF x UAF
AI/154 x T 60/78 (F21)	Ghana	UAF x UAF
**Parents**		
UPA134; SNK64; SNK614; SCA24; SNK625; NA33; T60/887; ICS89; SNK12; PA150; T60/78; T85/87; MAN15–12; AI/154;	Cameroon	UAF; LAF.;Tr
**Reference genotypes**		
Amelonado: Catongo; IFC5; MAT1–6 Contamana: SCA_11; SCA 6 Criollo: LAN 28b; LAN 14; B97 Curaray LCT 327; LCT 333; LCT57 Guyane: GU300; GU277; GU307 Iquitos: IMC48; IMC 87 Maranon: PA125; PA120; PA293; PA303 Nanay: NA435; NA184 Nacional: LCT 81; MO 76 Others controls: LCT 409; LCT 413; LCT362; EBC 125		

Tr= Trinitario, UAF=Forastero Haut Amazonien, LAF=Forastero Bas Amazonie.

### DNA purification and PCR amplification

Cocoa leaf samples were collected from each tree, placed in silica gels, and then quickly dehydrated in an oven at 120°C in the laboratory to prevent degradation during transport. Genomic DNA was extracted from the cocoa leaves following the method by [16] with slight modifications, and purified as described by Allègre et al. [17]. A total of 12 microsatellite markers, noted as mTcCIR, were selected. It is also noted that self-incompatibility microsatellite markers (mSI) were used. The primers mentioned in this study have already been described by Lanaud et al. [18,19] and then by Fouet et al. [20]. The characteristics such as nucleotide repeats, annealing temperature, chromosome number, forward and reverse primer sequences, allele size and number of alleles are provided in Table 2. These SSR markers were selected based on their high allelic polymorphism, ease of amplification, and reproducibility.

**Table 2:** Selected microsatellite markers and their characteristics.

Primer Name	Nucleotide repeats structure	Annealing Temperat. m(oC)	Chromosome localizaion	Forward primer (5’-3’) Reverse primer (5’-3’)	Size range (bp)	Number of alleles
mTcCIR 60	(CT)7(CA)20	51,0	2	CGCTACTAACAAACATCAAA AGAGCAACCATCACTAATCA	189-215	≥ 4
mTcCIR 292	(TC)12	56,0	2	TCCCCACAGCAACTACAA CTCTTCCCACCACCCA	236-255	≥ 8
mTcCIR 293	(AT)9	55,2	9	GAAAGGCCATATTGATGCT CTATTTCCACACTCAATTCCA	260-274	≥ 5
mTcCIR 294	(AG)14	54,4	9	GGGAGAGACACAGAGAGCTA GCCACTTTCTCCATCGT	112-136	≥ 9
mTcCIR 324	(CT)6	56,6	3	CGAAACTCTCTTCTTTCGCT GGCAGTGGGTTGGTTG	240-255	≥ 5
mTcCIR 336	(AGG)7	60,0	3	AGTGGGAGGAACAGTATGCG TAAACCGTGTCCACCAAACA	151-175	≥ 9
mTcCIR 359	(TC)7	56,4	4	TCGAGATACGCAAACGAA TGGACATTGCGAAAACC	187-190	≥ 4
mTcCIR 387	(GCT)7	59,1	6	CATGACCATTGCTTTCAACTCT AGCTGCCCGCGTTTT	212-224	≥ 4
mTcCIR 400	(ATT)5	56,0	9	TCAAAACGGGGAACAGA GTGTGCCGTTGTTTGGT	271-287	≥ 8
mSi 303	(AT)11	55,6	4	CAAGTCGTTGGGAGGG AAAGTTTCAATCCCATTTCC	179-259	≥ 16
mSi 458	(TA)11	59,3	4	GACACGAGATGTATCCTGACCA TGCAACCGTGAGCATTTTGT	263-343	
mSi 460	(TC)8	58,4	4	TGAGAACAAAGCCAAAGAAAGGA CCGAGACAAAGCCCAGAAG	96-176	≥ 17

PCR amplifications were performed using a PCT 200 gradient thermocycler. The PCR cycle consisted of an initial DNA denaturation for 5 minutes, followed by 10 successive “touch down” PCR cycles (starting hybridization temperature of 56°C decreasing by 1°C per cycle for 10 cycles): at 94°C for 30 seconds (initial DNA denaturation temperature), 56°C-46°C (primer hybridization temperature) for 45 seconds, and 72°C (strand elongation temperature during synthesis) for 45 seconds as well. This was followed by 25 PCR cycles at a fixed temperature of 46°C. This step was followed by a final elongation of the primer, at a temperature of 72°C for 8 minutes. The PCR products were then stored in a refrigerator at 4°C before analysis by capillary electrophoresis sequencing. At the end of the procedure, the temperature was maintained at 15°C. The amplification products from several 384-well plates (with different amplification sizes or fluorochromes) were then pooled into a single 384-well plate using a Biomek® NX robot (Beckman Coulter). Using the same robot, a program was used to distribute 2 µL from each well (containing multiple markers) into a new 384-well plate. A mixture of formamide and Ladder Liz 600 (Applied Biosystems) was added to each well (10µL). The fluorescence of the obtained DNA fragments was then revealed using a 16-capillary ABI 3500 XL sequencer (Applied Biosystems/ Hitachi). The electrophoresis data were analyzed using GENESCAN 3.7 software (Applied Biosystem Inc., USA) installed on a computer connected to the capillary sequencer. The SSR alleles were then analyzed in terms of fragment size, allelic designation, and internal standard using the Genemapper® v4.1 analysis software (Applied Biosystem). A dataset was generated in which each tree sample had a genotype corresponding to several loci. Data were exported using the Genotype Plot application and further analysis of raw data continued with Genemapper 4.1 software.

### Exclusion of off-types

318 hybrids and 15 parental genotypes were genotyped across 12 loci, after which duplicates and off-types were removed from our offspring analysis. Individuals displaying unique alleles were analyzed to assess genetic diversity and population structure.

After genotyping the descendants from various crosses, we performed detection of off-types (individuals that did not match the parental profiles). In total, 59 individuals, corresponding to an estimated rate of 18.55%, were identified as off-types.

### Comparison with reference clones

The reference genotypes used in this study were obtained from the cocoa plant genetic material database at the UMR-AGAP genotyping platform in Montpellier, France. Their alleles were compared to those generated from the population in this study (parents and descendants).

A profile for each reference sample was extracted from the database for the 12 SSR loci used in this study. The allele of each parent was compared to that of the reference genotype. Any descendant whose allele matches the reference genotype is considered compliant with the type. Any allele that does not match the reference genotype is considered off-type or a labeling error.

### Data analysis

Data were exported using the Genotype Plot application, and analysis continued with the Genemapper 4.1 software. Microsatellite markers were individually marked, and alleles were recorded by the presence of fragments of different lengths (polymorphic alleles) among individuals from each population. Only alleles that showed consistent amplification were used in the results analysis, and those presenting complicated profiles or too weak amplification were discarded.

### Analysis of genetic diversity

Genetic diversity was represented graphically to illustrate the variation among individuals. Power Marker V.4.03 software [21] was utilized to calculate allelic and genotypic frequencies. To assess the informativeness of the SSR markers, the polymorphism information content (PIC) for each marker was calculated using Nei’s formula [22]: PIC = 1 - Σ (Pi^2) from i = 1 to k, where k is the total number of alleles detected per locus and Pi is the frequency of the ith allele across all 360 cocoa genotypes, as calculated by [23]. The degree of polymorphism of each locus used in our study is represented by the PIC values with higher PIC values indicating greater discriminatory power among less informative loci. Based on genetic dissimilarity indices and a Neighbor-Joining method, a phylogenetic tree was constructed using DARWIN 6.0.1.4 software [24]. Additional genetic parameters calculated include: 1) Nei’s genetic parameters [25,26]: genetic distance, observed heterozygosity (Ho), and genetic diversity, often referred to as expected heterozygosity (He); 2) Genetic polymorphisms [27]were calculated by the effective number of alleles per locus (Ne); 3) Wright’s fixation indices (F-statistics) were measured at different hierarchical levels, according to Weir and Cockerham [28]: F_IS (allele correlation within individuals of a population), F_ST (allele correlation among individuals of a population relative to the total population), and F_IT (allele correlation within individuals representing “inbreeding”).

### Population genetic structure

Two methods were employed to infer the genetic structure of the population under study. Initially, a distance-based model using the dissimilarity matrix was computed using the Neighbor-Joining method [29], as implemented in DARWIN 6.0.14 software. This assessed the genetic structure among the 360 representative genotypes of T. cacao, initially evaluated for congruence with previously identified genetic groups [8]. Subsequently, a phylogenetic analysis including both collected and reference genotypes was constructed using the Neighbor-Joining method with 500 bootstrap repetitions to evaluate the uncertainty of the tree structure. Additionally, a Bayesian model was applied using STRUCTURE 2.3.4 software [28]. K groups ranging from 2 to 10 were tested with a burn-in period of 100,000 iterations followed by 500,000 Markov Chain Monte Carlo repetitions, with at least 15 repetitions per K. The optimal K value, indicating the presumed level of underlying structure, was determined using the method described by Evanno et al. [30] and the Structure Selector [31]. This software also incorporates the CLUMPAK program [32], which combines several features of existing tools to post-process STRUCTURE results by incorporating calls to CLUMPP [33] and DISTRUCT [34].

## Results

### Genotype determination

**Fig 1:**
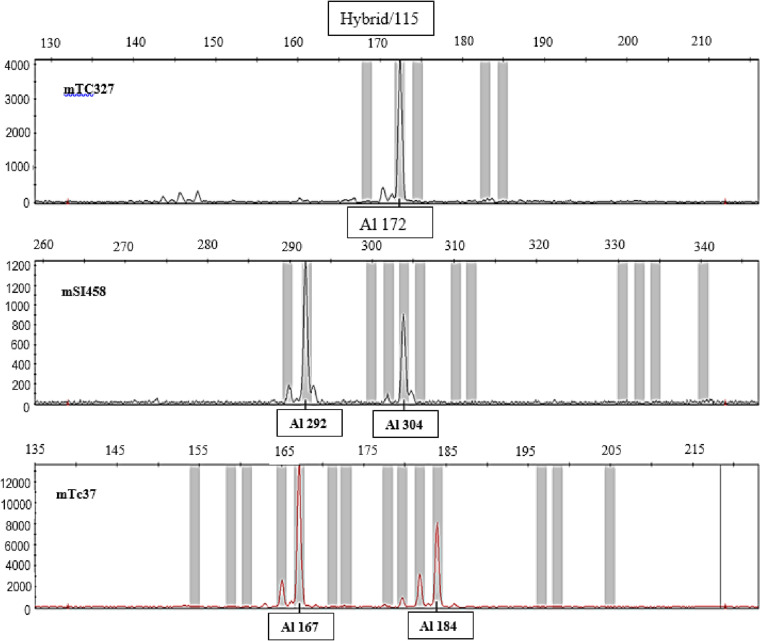
Reading the amplifications on Genemapper® v4.1 from Applied Biosystem. Al = allele.

The genotypes of individuals amplified with microsatellite markers and revealed on the capillary sequencer were analyzed using Genemapper® software v4.1 (Applied Biosystems). Multiple alleles were identified at each of the 12 loci. The fragment sizes were automatically calculated. The alleles corresponding to each fragment were then identified using Genemapper version 4.1; this was done after defining the size range for each marker locus (minimum and maximum number of base pairs). By graphically viewing the alleles in Genemapper (curves), they exhibit one or two peaks (Fig 1), as expected when using co-dominant markers such as microsatellites: genotypes yielding two peaks (2 alleles) correspond to heterozygous individuals, while those with a single peak (one allele) are homozygous. For some samples (genotypes), curves displaying more than two peaks were recorded; only the two highest peaks were considered. Only alleles that demonstrated consistent amplification were used in the analysis of results, and those with profiles that were difficult to interpret or weakly amplified were excluded from the analyses.

### Analysis of genetic diversity

All the markers employed in this study have previously been used in research focused on genetic diversity, mapping, population identity analysis, and even the evaluation of self-incompatibility in cocoa trees [8,13,14,19,35,36] (Table 3). These microsatellite markers have been mapped in various cocoa populations. The total number of alleles detected across the 12 loci was 55 for the parents and 72 for the offspring, with an average of 4.58 alleles per locus for the parents and 6 for the offspring.

**Table 3:** Genetic Parameters of SSR Markers. Number of Alleles per Locus, Observed Heterozygosity (H_obs) and Expected Heterozygosity (H_exp), Polymorphic Information Content (PIC), Wright’s F-Statistics (F_IT, F_IS, F_ST), Hardy-Weinberg Equilibrium Probabilities (HW), and Shannon’s Diversity Index.

Primer Name	Number of alleles	H obs	H exp	PIC	FIS	FIT	Hw Prob	Shannon index	Genetic diversity
**Parents (a)**									
mSI303	6	0.28	0.81	0.83	0.66	0.1	0.80	1.61	0.84
mSI460	9	0.67	0.89	0.85	0.24	0.1	0.88	2.08	0.86
mTc294	4	0.40	0.40	0.50	-0.03	0.03	0.39	0.73	0.56
mTc336	5	0.60	0.79	0.86	0.23	0.27	0.78	1.52	0.87
mTc359	4	0.53	0.71	0.81	0.24	0.1	0.71	1.26	0.84
mTc60	8	0.70	0.89	0.82	0.20	0.25	0.87	1.97	0.84
mSi458	7	0.40	0.78	0.79	0.48	0.03	0.77	1.61	0.82
mTc293	4	0.15	0.58	0.69	0.73	0.62	0.58	1.03	0.72
mTc400	1	0.00	0.00	0.31	nd	1.00	nd	0.00	0.39
mTc292	3	0.67	0.49	0.61	-0.40	0.67	0.47	0.76	0.67
mTc324	2	0.50	0.52	0.67	0.02	0.55	0.51	0.69	0.72
mTc387	2	0.13	0.33	0.39	0.59	0.8	0.33	0.50	0.43
**Mean**	**4.58**	**0.42**	**0.6**	**0.68**	**0.64**	**0.38**	**0.27**	**1.15**	**0.71**
**Hybrids (b)**									
mSI303	10	0.74	0.79	0.85	0.06	0.06	0.79	1.74	0.86
mSI460	11	0.89	0.86	0.94	-0.03	0.12	0.86	2.14	0.94
mTc294	6	0.34	0.40	0.58	0.15	0	0.39	0.84	0.60
mTc336	6	0.85	0.77	0.88	-0.10	0.3	0.77	1.56	0.89
mTc359	4	0.66	0.65	0.81	-0.02	0.06	0.65	1.18	0.83
mTc60	10	0.71	0.82	0.90	0.13	0	0.82	1.87	0.91
mSi458	8	0.58	0.77	0.90	0.24	0.03	0.77	1.69	0.90
mTc293	4	0.44	0.66	0.80	0.34	0.47	0.66	1.21	0.82
mTc400	5	0.18	0.18	0.42	-0.01	0	0.18	0.43	0.44
mTc292	4	0.32	0.39	0.63	0.20	0.02	0.39	0.68	0.69
mTc324	2	0.59	0.50	0.64	-0.18	0.5	0.50	0.69	0.69
mTc387	2	0.20	0.18	0.30	-0.11	0.9	0.18	0.32	0.35
**Mean**	**6**	**0.54**	**0 .58**	**0.72**	**0.06**	**0.2**	**0.58**	**1.2**	**0.74**

### Genetic parameters analysis

Table 3 presents the values of genetic parameters used to analyze the polymorphism of the 12 SSR markers across all 360 DNA samples (hybrids, parents, and reference clones). Among the hybrid offspring, the total number of alleles detected across the 12 loci was 72 within the hybrid families of the regional variety trial, averaging 6.00 alleles per locus. The number of alleles per locus ranged from 11 alleles at locus mSI460–2 alleles at loci mTcCIR387 and mTcCIR324 (Table 3a). Observed heterozygosity (Ho) for the hybrid families ranged from 0.89 at mSI460 to 0.18 at mTcCIR400, with an average of 0.54 across all 12 loci. Overall, genetic diversity is determined by the likelihood that two randomly selected alleles ²from the population are different. Additionally, expected heterozygosity (H exp) among the offspring varied from 0.86 at mSI460 to 0.18 at mTcCIR400 and mTcCIR387, with an average of 0.58 across the 12 loci. The Polymorphic Information Content (PIC) ranged from 0.94 at mSI460 to 0.30 at mTcCIR387 and 0.42 at mTcCIR400, with an average of 0.57 (Table 3a). Almost all PIC values were above 0.60, averaging 0.72, indicating highly polymorphic markers. The marker mSI460 (0.94) is the most informative. The degree of polymorphism ranged from 0.63 (mTcCIR292) to 0.90 (mTcCIR60). Markers mTcCIR387 (0.30) and mTcCIR400 with PICs less than 0.5 are consequently less informative.

The effect of inbreeding (relative lack of heterozygosity) within the entire agricultural population was estimated by FIS values for each locus, ranging from -0.18 (mTcCIR324) to 0.34 (mTcCIR293), thus indicating a variable rate of self-fertilization throughout the studied population. FIT values for the entire population showed substantial variation between loci from mTcCIR400 to mTcCIR387 (0.00–0.90).

The average values of Hardy-Weinberg probabilities [37] for the entire population were estimated for each locus. A deviation from Hardy-Weinberg equilibrium is observed for most loci, especially for mTcCIR387, mTcCIR400, mTcCIR292, and mTcCIR294, indicating that allele transmission from one generation to the next in all hybrid families in this study is influenced by evolutionary forces. Thus, the Shannon diversity index, which measures genotypic diversity (rather than allelic diversity), varied from 0.32 to 2.14 (Table 3), indicating a low level of diversity across the studied population.

Among the parents, the average number of alleles per locus for the 12 markers was 4.59. The highest number of alleles was detected at locus mSI460 (9 alleles) and the lowest at loci mTcCIR387 and mTcCIR324 (2 alleles). A heterozygote deficit (HE>HO) was recorded for almost all markers used except mTcCIR400 (0.00); mTcCIR294 (0.40), and mTcCIR324 (0.44). Furthermore, observed heterozygosity varied from 0.00 (mTcCIR400) to 0.67 (mTcCIR294 and mTcCIR292). PIC values ranged from 0.85 (mSI460) to 0.31 (mTcCIR400), with an average of 0.68. Markers mSI460 and mTcCIR336 are the most informative, with respective values of 0.85 and 0.86. Markers mTcCIR387 (0.39) and mTcCIR400 (0.31) with PICs less than 0.5 are consequently less informative.

### Population structure

**Fig 2:**
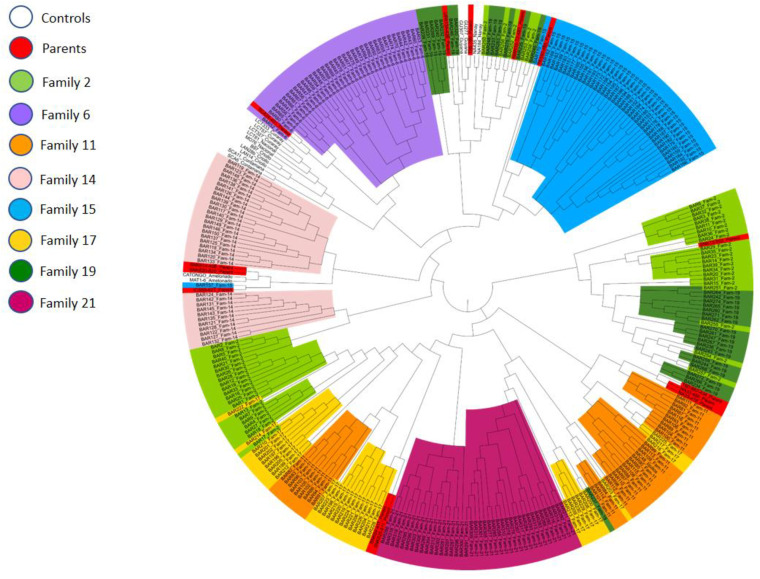
Neighbor-joining tree of individuals in the Theobroma cacao, based on the allelic dissimilarity calculated from SSR data set by Darwin software.

A phylogenetic analysis was conducted using Darwin software version 6.0.14 [29] to assess the genetic similarities among different families (various crosses) and control samples representing the various genetic groups described for the cacao tree [8]. This analysis helped confirm the genetic structuring of the populations according to known genetic lineages and provided insights into the genetic relationships and diversity within the trial (Fig 2).

### Phylogenetic tree analysis

In all, we used 12 SSR markers for our study. Apart from the fact that some presented a low amplification they had again a lot of missing data (DM). it was a question of minimizing missing data. To this, we remove three SSR markers mSI 303. mTCCIR 292; mTCCIR324 because they had a lot of DM.

A phylogenetic tree was draw up reflecting the genetic distance calculated on the basis of percentage of alleles share between the different trees analyzed using the Neighbour joining method with Darwin software (Fig 2). The length of the tree branches is proportional to their genetic distance. We observed that, with the exception of a few individuals from certain families, the majority of the offspring are grouped with their parents and the reference clones, demonstrating a high genetic similarity among them and the greatest genetic divergence among the reference genotypes.

**Fig 3:**
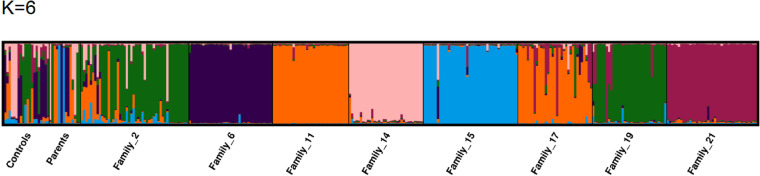
Homogeneous and distinct structure of 335 Barombi-kang cocoa genotypes obtained with structure software using SSR (K= 6). Each color represents a family and the inviduals represented within it.

. Most offspring result from crosses between Upper Amazonian and Trinitario; or between Lower Amazonian and Trinitario.In these results, most of the genotypes share alleles with their parents (about 90%). Of the 10 genetic groups identified by Motamayor et al., [8], our crosses yielded six genetic groups: Amelonado, Iquitos, Guyana, Maranon, Nanay, and Contamana. The Amelonado group represented nearly half of the individuals in our offspring (T60/887 x ICS89; T60/78 x T85/88; SNK625x NA33; UPA x SNK64), and the rest are Trinitario, which are crosses between Amelonado and Upper Amazonian Forastero. We did not encounter any Criollo, Purus, Curaray, or Nacional groups. At K=6 we also observe homogeneous and distinct population structure. Each horizontal bar represents a family and the individuals represented within it. The families UPA*SNK 64 and MANS15/2*T85/799 have very similar alleles. Since our hybrid families are full sibling, we find many similarities between individuals from different families (alleles) within the same population (Fig 3).

## Discussion

### SSR marker diversity, genetic relationship among cocoa genotypes, verification of material compliance

The verification of plant material compliance in Cameroon is an important step in the cocoa tree selection and productivity improvement process. These SSR markers, developed by Lanaud et al. [18], have proven to be highly polymorphic and are extensively used to assess labeling errors and duplicates in national and international gene banks. The use of multilocus microsatellite profiles is significantly more accurate because genotypes can have different characteristics, which helps avoid labeling errors. Specifically, identical genotypes can match in multilocus microsatellite profiles. Only the genotypes for which two peaks are obtained (2 alleles) correspond to heterozygous individuals, and those for which one peak is observed (a single allele) are homozygous. For some samples (genotypes), curves with more than two peaks have been recorded; only the two highest peaks were retained. The error percentage of individuals classified according to the genetic groups of Motamayor et al. [8] is 18.55%, or 59 individuals out of 360; these detected labeling errors can be due to either pollen transfer during artificial pollinations, contamination, or a mix-up of plant material in the Barombi-Kang regional variety trial. These results align with those of other studies on cocoa germplasm collections that have detected error rates of about 40% [38–42].

Allelic diversity in this study was observed with an average of 6 alleles per locus (total of 72 alleles) among descendants, and 4.58 alleles per locus (total of 55 alleles) among parents. These findings are comparable to those reported in Ecuador, Nicaragua, and the Dominican Republic by Zhang et al., [43]; Sereno et al., [35], where they found respectively 4.20 alleles per locus (total of 63 alleles) and 4.45 alleles per locus (total of 49 alleles). Similar results were also observed by Loor et al., [14] in collections along the Ecuadorian coast, with 4.22 alleles per locus and a total of 169 alleles, although here the total number of alleles is significantly higher. In Bolivian cocoa collections [44], the values were lower, at 3.7 alleles per locus with a total of 75 alleles. An exception was made in the field study and cocoa germplasms in Cameroon by Efombagn et al., [13], which showed a very high allelic diversity (9.41 alleles per locus for a total of 125 alleles). These results corroborate those obtained by Irish et al., [45] in Puerto Rico; Bozar et al., [41] in the Dominican Republic; Bidot et al., [42] in old populations introduced to Cuba; Fouet et al., [46] in native collections in Ecuador. This result confirms that the markers used for our study were more informative.

Moreover, the SSR markers we used had an average PIC value of 0.68 in parents and 0.60 in hybrid families, indicating that these markers are very informative in our study. The most polymorphic marker in both parents and descendants is mSI460. Indeed, Botstein et al., [47] reported that a PIC value greater than 0.5 is considered indicative of a highly informative marker, while a value between 0.5 and 0.25 corresponds to a moderately informative marker. Previous studies by Efombagn et al., [13] found a PIC value of 0.59 in 265 cocoa genotypes. Tekeu et al., [48] found an average PIC value of 0.69 in 17 wheat cultivars.

As indicated by the F-statistics, the genetic diversity in our study is characterized by a high level of heterozygosity. Heterozygosity levels in hybrids (Hexp = 0.6) and in parents (Hexp = 0.58) were detected. The higher level of heterozygosity among descendants could be explained by the cocoa tree’s self-incompatibility system, which varies according to genetic differences. Trinitarios and upper Amazonian Forasteros are generally self-incompatible, while Amelonados or lower Amazonian Forasteros are self-compatible [49–51]. The high genetic diversity indicates substantial mixing levels within our population’s genetic pool. The genetic diversity observed in hybrids and parents is lower than that reported for a population in the Ucayali Valley in Peru (Hexp = 0.74; [35]) and Ghana (Hexp = 0.74; [52]), but higher than that described in Nicaragua (Hexp = 0.476; [53]), Brazil (Hexp = 0.497; [36], and by Loor et al., [14] (Hexp = 0.496).

In examining the compliance of the material used, these genotypes closely match (at least 90%) their respective parents. Samples show a high level of variation. Among them, some homozygotes have been identified despite the hybrid nature of most. These genotypes showed a high level of introgression of Trinitario alleles and share the same alleles for several loci between offspring, with parents, and with the reference genotypes in our study.

The phylogenetic tree in this study explains the predominant diversity and detected mixing among parents and descendants. The degree of genetic diversity could thus be due to the diversified introduction of material into the country. Understanding the genetic diversity, population structure, and verification of plant material compliance will greatly enhance the selection of elite cocoa trees. However, the genotypes used as parents in this study were selected for hybrid variety development. These clones are known to be practically tolerant to brown rot [54–56].

The software Structure (Pritchard et al. 2000) was extensively used for Bayesian classification analysis, which assigns groups of individuals based on population membership coefficients and determines the degree of mixing or allelic contributions in a population [57,58,59]. When the 318 descendant individuals and 15 parental genotypes were analyzed with the representative genotypes of the ten genetic groups previously reported by Motamayor et al. [8], each individual had the highest membership coefficient to one of the ten genetic groups. Six of the 10 genetic groups were represented in this genetic material, and a majority of the genotypes corresponded to Amelonado, Iquitos, Guyana, Maranon, Nanay, and Contamana. The highest membership coefficient for the majority corresponded to the Amelonado group.

The structure analysis was conducted with the inclusion of representative genotypes corresponding to the ten genetic groups. Significant levels of mixing were detected in our population, however, only nine groups were identified.

After removing duplicates and mislabeling only 59 individuals in the study were identified as off-types compared to the 360 individuals analysed.

## Conclusion

This study effectively mapped the genetic diversity and verified plant material compliance of cocoa genotypes in the Barombi-Kang Regional variety trial, highlighting substantial genetic variability and significant labeling inaccuracies. The high levels of genetic diversity and polymorphism detected among the genotypes underscore a strong genetic foundation, which is critical for the advancement of resilient and productive cocoa varieties. Addressing the identified labeling errors is crucial for ensuring the accuracy of breeding programs. Implementing stringent verification protocols will enhance the reliability of these programs, contributing positively to the sustainable development of the cocoa industry in Cameroon.
